# Analysis of uncorrected near visual acuity after extended depth-of-focus AcrySof® Vivity™ intraocular lens implantation

**DOI:** 10.1371/journal.pone.0277687

**Published:** 2022-11-28

**Authors:** Sohee Jeon, Ayoung Choi, Hyunggoo Kwon

**Affiliations:** Keye Eye Center, Seoul, Republic of Korea; Universidad de Monterrey Division de Ciencias de la Salud, MEXICO

## Abstract

A newly developed extended-depth-of-focus AcrySof^®^ Vivity™ intraocular lens (IOL), which has a wavefront-shaped anterior surface, has shown a promising outcome in minimizing dysphotopsia, the biggest issue after diffractive type IOL implantation. On the contrary, relatively low uncorrected near visual acuity (UNVA) has been raised as a demerit of this IOL. However, there is only limited information about the UNVA after Vivity implantation. In the present study, we compared the uncorrected distant and intermediate visual acuity (UDVA and UIVA) and UNVA according to the range of refractive error (RE) from 91 eyes from 91 patients implanted with Vivity IOL. Then we assessed the biometric factors for their association with UNVA from 66 eyes with a RE within ± 0.25 D. The UDVA was worst in eyes with RE < -0.50 D (0.17 ± 0.21), which was significantly worse than in any other group (*P* < 0.001 for every analysis). The UIVA was worst in eyes with RE of 0.25 to 0.50 D (0.35 ± 0.07 D), which was significantly worse than in eyes with RE of -0.50 to -0.26 D (*P* = 0.020) and in eyes with RE of -0.25 to -0.01 D (*P* = 0.028). The UNVA was worst in eyes with RE of 0.25 to 0.50 D (0.40 ± 0.14 D), which was significantly worse than in eyes with RE of -0.50 to -0.26 D (*P* = 0.022), which suggests that the extent of monovision should be limited up to -0.50 diopter. On univariate analysis for UNVA in eyes with a RE within ± 0.25 D, the anterior chamber depth (*R* = 0.257; *P* = 0.037) and pupil size (*R* = 0.451; *P* < 0.001) had a statistically significant relation to UNVA, while multivariate analysis showed the pupil size (β = 0.451; *P* < 0.001) as the sole indicator, suggesting eyes with a small pupil size might receive a UNVA benefit.

## Introduction

Given the mounting public desire for glasses-free living, and with improvements in the quality of modern intraocular lenses (IOLs), implantation of multifocal IOLs (MIOLs) has increased substantially in recent decades. Different types of MIOLs vary in their optical design, but the diffractive type provides the highest amount of near addition through stepwise induction of focus. The abrupt divisions between each zone of focus inevitably produce visual complications, such as dysphotopsia, and reduce contrast sensitivity [1]. To solve this issue, the extended depth-of-focus AcrySof^®^ Vivity™ lens has developed, which has a wavefront-shaped anterior surface that produces a continuous extended focal range, minimizing postoperative dysphotopsia [2–5].

Studies have shown good uncorrected distant visual acuity (UDVA) and uncorrected intermediate visual acuity (UIVA) but relatively poor uncorrected near visual acuity (UNVA) after implantation of a Vivity lens, when compared with previous diffractive type MIOLs [2–6]. However, the postoperative UNVA varies considerably from patient to patient: from J7 to J1. It would be helpful to be able to predict the postoperative UNVA to guide IOL selection for patients who have a strong need for UNVA but who are afraid of postoperative visual complications. We conducted this retrospective study to evaluate the predictive factors for UNVA after Vivity implantation.

## Patients and methods

We performed a retrospective review of the records of patients who underwent phacoemulsification and lens implantation using the Vivity IOL between May 15, 2021, and December 31, 2021, at Keye Eye Center, Seoul, Korea. All procedures were performed by 2 experienced surgeons (HK and SJ) through a standard sutureless 2.2-mm microincision. Only one eye was randomly selected when both eyes were eligible. Eyes with poor retinal function, preoperative corrected distance visual acuity (CDVA) less than 20/40, previous corneal or vitreoretinal surgery, intraoperative capsular damage, or any postoperative complication were excluded from analysis. The institutional review board (IRB)/ethics committee of Keye Eye Center approved the study (IRB number P12360228-002) and waived the requirement for informed consent because of its retrospective nature. The study protocol adhered to the tenets of the Declaration of Helsinki.

### Outcome measures

In most patients, the IOL power was selected to target emmetropia. We chose the first available negative-power IOL using the Kane formula based on the biometric data from IOLMaster 700 (Carl Zeiss Meditec AG, Jena, Germany). At our institution, we implant toric IOL for patients with a corneal astigmatism higher than 0.50 D.

Following preoperative ocular data was collected; axial length (AL), lens thickness (LT), anterior chamber depth (ACD), horizontal corneal diameter (HCD), mean keratometry (Km), corneal astigmatism (Ka), pupil size measured with two partial coherence interferometry devices (Anterion; Heidelberg Engineering, Heidelberg, Germany and IOLMaster 700). The high order aberrations and pupil size were collected from Pentacam Scheimpflug System (Oculus Optikgeräte GmbH). Pupil size was measured under photopic illumination. The pupil size from Anterion was used as a primary data throughout the analysis.

Objective and subjective refraction in spherical equivalent (oSE and sSE), and monocular UDVA, CDVA, UIVA, and UNVA were assessed at postoperative months 1, 2, and 6. Objective refraction was measured using an autorefractor (ARK-1; NIDEK Co., Ltd., Tokyo, Japan). Subjective refraction was measured by retinoscopy using a retinoscope (Keeler Ltd., Windsor, UK) and a retinoscopy rack lens set. Visual acuity was measured using decimal fractions and converted into LogMAR for statistical analysis. Near- and intermediate visual acuity was measured using the Sloan ETDRS Format Near Vision Chart 3 with 100% contrast under photopic conditions (167 candelas [cd]/m^2^) at 40 and 66 cm. We assessed contrast sensitivity using the CGT-2000 instrument (Takagi Seiko Co. Ltd., Nagano-Ken, Japan) at postoperative month 2 or 6. The quality of vision was evaluated by Strehl ratio and Area ratio using OPD-Scan III (NIDEK Co. Ltd., Aichi, Japan).

### Statistical analysis

All computations were made using standard software (SPSS version 15.0 for Windows; SPSS Inc., Chicago, IL). Descriptive data are expressed as the mean ± standard deviation unless otherwise specified. Analysis of variance (ANOVA) was used to compare 3 or more data points. The Bonferroni test was used for post-hoc analysis. Agreement of measurements from the 3 ddevices was assessed with the method previously described by Bland and Altman [7], who recommend plotting the differences between measurements (y-axis) against their mean (x-axis) and calculating the 95% limits of agreement (LoA) as the mean ±1.96 standard deviation (SD) of the differences between the 2 measurements techniques using R Project (http://www.r-project.org/). Intraclass correlation coefficient (ICC) was used to compare the pupil size as measured by different devices. The Bonferroni correction was applied for the correlation analysis between pupil size from each device and UNVA. Since we performed three analyses, the value for statistical significance was set as 0.017. The Pearson correlation coefficient was used to assess associations between continuous variables, according to normality of distribution. Independent variables that showed significance on univariate analyses were included as independent covariables on multivariate analyses (multiple regression analysis). All *P* values were 2-sided, and significance was defined as a *P* value less than 0.05.

## Results

We assessed data from 91 eyes from 91 patients that were implanted with Vivity IOL. The patients’ baseline demographics are summarized in Table 1. The mean patient age was 59.80 ± 5.22 years, and 31.9% of patients were men. The preoperative UDVA and UNVA were 0.38 ± 0.23 and 0.45 ± 0.21 and improved to 0.01 ± 0.05 and 0.22 ± 0.11 postoperatively. The mean postoperative oSE and sSE was -0.21 ± 0.37 and -0.19 ± 0.24.

**Table 1:** Preoperative and postoperative data of enrolled patients (n = 91).

Parameter	Value ± SD
**Age, yrs**	59.80 ± 5.22
**Sex, male (%)**	29 (31.9)
**Preoperative visual acuity**	
**UDVA, LogMAR**	0.38 ± 0.23
**CDVA, LogMAR**	0.04 ± 0.09
**UNVA, LogMAR**	0.45 ± 0.21
**Preoperative oSE, D**	0.61 ± 1.68
**Axial length, mm**	23.60 ± 0.78
**Anterior chamber depth, mm**	3.23 ± 0.31
**Lens thickness, mm**	4.52 ± 0.27
**Mean keratometry, D**	43.02 ± 1.63
**Astigmatism, D**	-0.46 ± 0.26
**Pupil size, mm**	4.42 ± 0.68
**Corneal diameter, mm**	11.75 ± 0.46
**High order aberration, μm**	0.07 ± 0.02
**Spherical aberration, μm**	0.02 ± 0.01
**Postoperative visual acuity**	
**UDVA, LogMAR**	0.01 ± 0.05
**CDVA, LogMAR**	0.00 ± 0.01
**UIVA, LogMAR**	0.11 ± 0.11
**UNVA, LogMAR**	0.22 ± 0.11
**Postoperative oSE, D**	-0.21 ± 0.37
**Postoperative sSE, D**	-0.19 ± 0.24

CDVA = corrected distance visual acuity; D = diopter; oSE = objective refraction in spherical equivalent; sSE = subjective refraction in spherical equivalent; UDVA = uncorrected distance visual acuity; UIVA = uncorrected intermediate visual acuity; UNVA = uncorrected near visual acuity.

**Fig 1:**
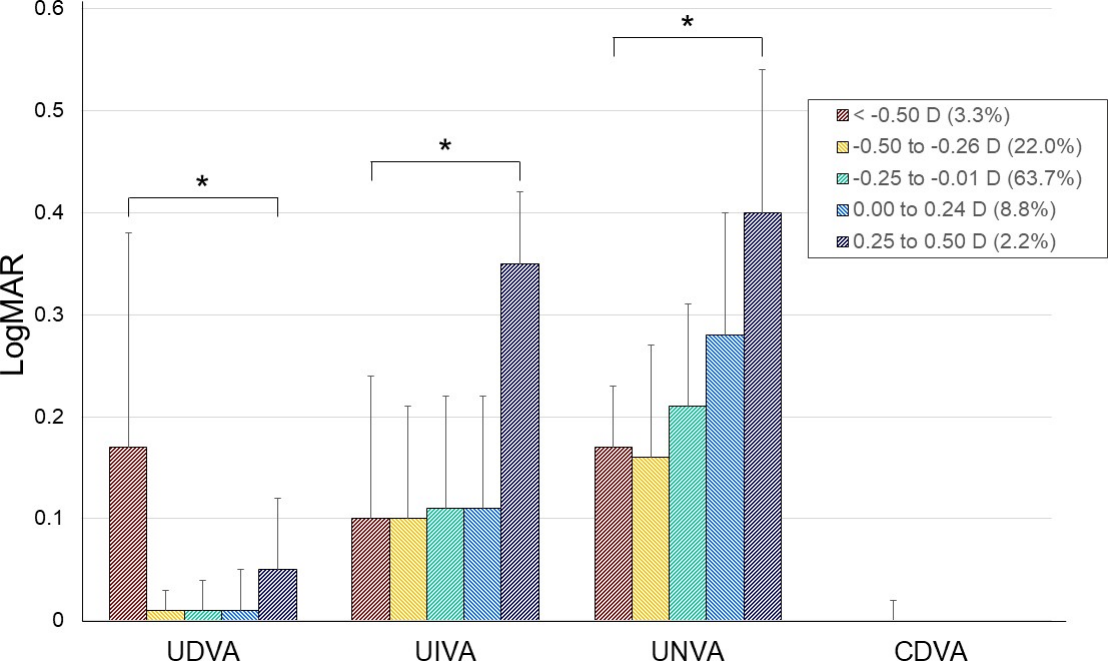
Postoperative visual acuity according to the range of subjective refraction. * *P* < 0.05.

Among 91 eyes, 3 (3.3%), 20 (22.0%), 58 (63.7%), 8 (8.8%), and 2 (2.2%) eyes showed postoperative sSE of < -0.50, -0.50 to -0.26, -0.25 to -0.01, 0.00 to 0.24, and 0.25 to 0.50 D. Fig 1 shows the visual acuities of enrolled eyes according to their sSE. The UDVA, UIVA and UNVA were statistically significantly different between groups (*P* < 0.001, *P* = 0.043 and *P* = 0.007, ANOVA test), but CDVA was not (*P* = 0.418, ANOVA test). The UDVA was worst in eyes with sSE < -0.50 D (0.17 ± 0.21); this value was significantly worse than in any other group (*P* < 0.001 for eyes with sSE -0.50 to -0.26 D [UDVA, 0.01 ± 0.02], *P* < 0.001 for eyes with sSE of -0.25 to -0.01 D [UDVA, 0.01 ± 0.03], *P* < 0.001 for eyes with sSE of 0.00 to 0.24 D [UDVA, 0.01 ± 0.04], *P* = 0.038 for eyes with sSE of 0.25 to 0.50 D [0.05 ± 0.07]; post-hoc analysis using Bonferroni test). The UIVA was worst in eyes with sSE of 0.25 to 0.50 D (0.35 ± 0.07 D); this value was significantly worse than eyes with sSE of -0.50 to -0.26 D (0.10 ± 0.14; *P* = 0.020) and eyes with sSE of -0.25 to -0.01 D (0.11 ± 0.11; *P* = 0.028). The UNVA was worst in eyes with sSE of 0.25 to 0.50 D (0.40 ± 0.14 D); this value was significantly worse than eyes with sSE of -0.50 to -0.26 D (0.16 ± 0.11; *P* = 0.022).

**Fig 2:**
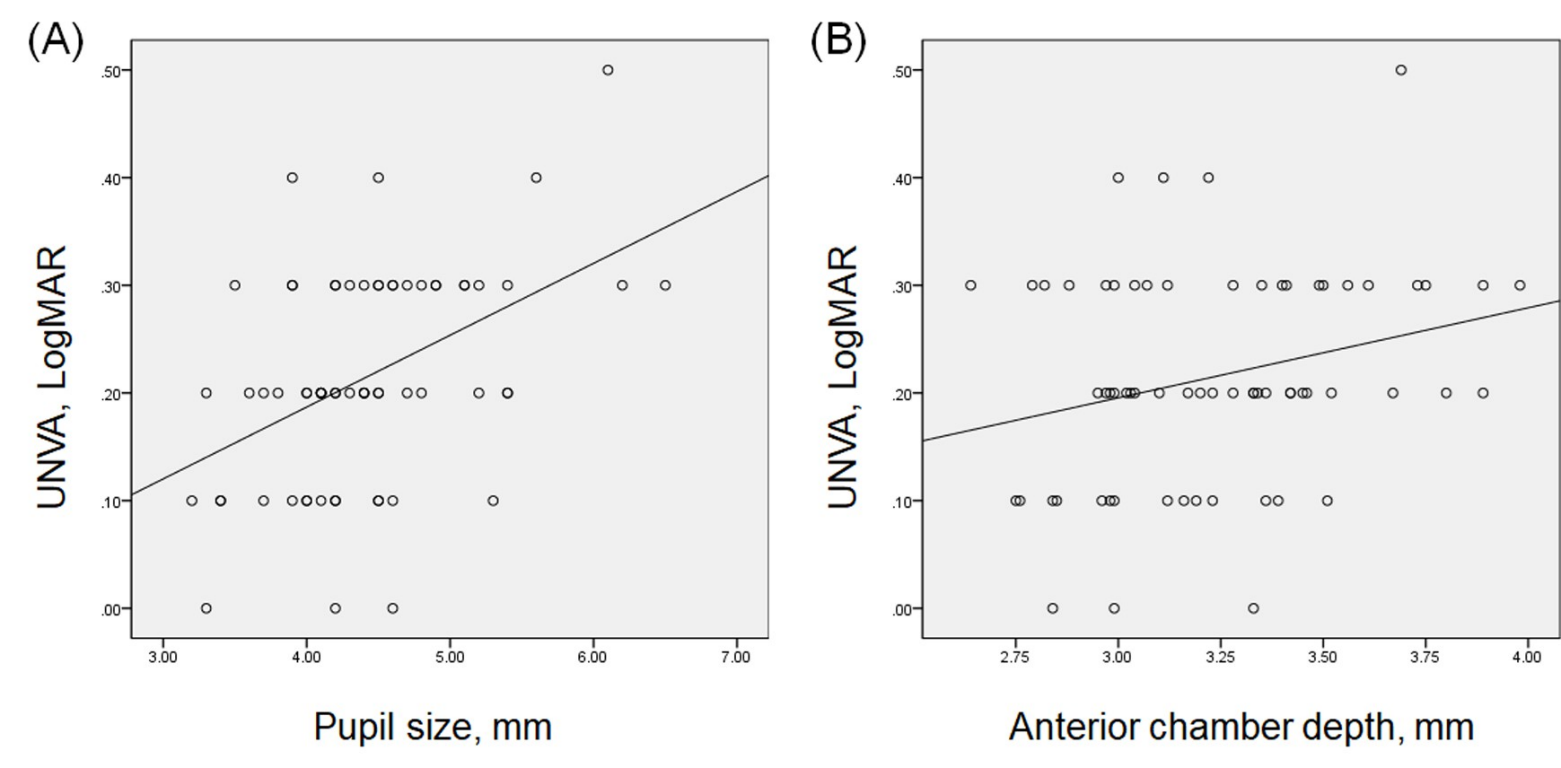
Dot graphs showing correlation between uncorrected near visual acuity (UNVA) and (A) pupil size (*R* = 0.451; *P* < 0.001) and (B) anterior chamber depth (*R* = 0.257; *P* = 0.037).

We selectively analyzed the eyes that had an sSE within ± 0.25 D to evaluate the factors associated with UNVA in emmetropic Vivity-implanted eyes (n = 66; Table 2). On univariate analysis, the pupil size (*R* = 0.451; *P* < 0.001, Fig 2A) and ACD (*R* = 0.257; *P* = 0.037, Fig 2B) had a statistically significant relation to UNVA in emmetropic Vivity-implanted eyes. Multivariate analysis showed the pupil size (β = 0.451; *P* < 0.001) as the sole indicator for postoperative UNVA in emmetropic Vivity-implanted eyes.

**Table 2:** Univariate and multivariate analysis for the UNVA in emmetropic eyes.

	Univariate analysis		Multivariate analysis	
	R	P	β	P
**Age**	-0.190	0.127		
**Axial length**	0.142	0.254		
**Anterior chamber depth**	0.257	0.037		
**Lens thickness**	0.060	0.631		
**Mean keratometry**	-0.046	0.715		
**Astigmatism**	-0.096	0.445		
**Pupil size**	0.451	< 0.001	0.451	< 0.001
**Corneal diameter**	0.158	0.204		
**High order aberration**	-0.089	0.476		
**Spherical aberration**	-0.006	0.964		
**Tilt**	-0.096	0.297		
**Coma**	-0.013	0.885		
**Subject refractive error**	0.101	0.419		

As pupil size can be measured by multiple modalities, we collected information that came from the Anterion, IOLMaster 700, and Pentacam (Table 3). The measurement of pupil size from each device showed good correlation; ICC of 0.839 (95% confidential interval 0.758–0.896, *P* < 0.001) when analyzed with intraclass correlation coefficient. The Bland-Altman analysis showed bias of 0.68 (95% LoA; 0.58–0.78) for Anterion vs IOLMaster, 1.59 (95% LoA; 1.47–1.70) for Anterion vs Pentacam, and 0.91 (95% LoA; 0.82–1.00) for Pentacam vs IOLMaster. These results suggest a consistent trend that the measurements were largest with Anterion, followed by IOLMaster and Pentacam, and those values from each parameter are not interchangeable. While the pupil size detected by Anterion and IOLMaster 700 showed a significant correlation with UNVA (*R* = 0.451; *P* < 0.008 for Anterion and *R* = 0.324; *P* = 0.008 for IOLMaster), the measurement from Pentacam was not (*R* = 0.176; *P* = 0.158).

**Table 3:** Pupil size measurements from various machines.

	Pupil size (range), mm	Correlation with UNVA	
		R	P
**Anterion**	4.42 ± 0.68 (3.20–6.50)	0.451	< 0.001
**IOLMaster 700**	3.74 ± 0.63 (2.70–5.70)	0.324	0.008
**Pentacam**	2.87 ± 0.53 (2.03–4.52)	0.176	0.158

UNVA = uncorrected near visual acuity

Then we analyzed the correlation of pupil size with other visual parameters to evaluate the potential effect of pupil size on other visual function. There was no correlation between pupil size and UDVA, UIVA, contrast sensitivity in mesopic or photopic condition (calculated using the area under the log contrast sensitivity function), Strehl ratio, or Area ratio (Table 4).

**Table 4:** Correlation between pupil size and visual functions.

	Pupil size of IOLMaster 700		Pupil size of Anterion	
	R	P	R	P
**UDVA**	-0.022	0.859	0.038	0.759
**UIVA**	0.017	0.890	0.201	0.105
**Mesopic AULCSF**	-0.109	0.452	-0.186	0.195
**Photopic AULCSF**	0.030	0.835	-0.080	0.581
**Strehl ratio**	-0.101	0.432	0.039	0.761
**Area ratio, 4 mm**	-0.132	0.329	0.061	0.651
**Area ratio, 5mm**	-0.087	0.497	0.040	0.757

AULCSF = area under the log contrast sensitivity function; UDVA = uncorrected distant visual acuity; UIVA = uncorrected intermediate visual acuity

Table 5 shows the characteristics of patients with excellent UNVA, defined as equal to or better than 0.10. Eyes with excellent UNVA had a smaller ACD (3.07 ± 0.24 vs 3.29 ± 0.32; *P* = 0.013), smaller astigmatism (-0.33 ± 0.17 vs -0.51 ± 0.27; *P* = 0.014), and smaller pupil size (4.06 ± 0.55 vs 4.55 ± 0.68; *P* = 0.011).

**Table 5:** Clinical characteristics of patients with excellent UNVA among emmetropic Vivity-implanted eyes.

	UNVA ≤ 0.10 (n = 17)	UNVA > 0.10 (n = 49)	P value
**Age**	59.59 ± 4.80	58.77 ± 4.93	0.558
**Axial length**	23.51 ± 0.65	23.63 ± 0.83	0.610
**Anterior chamber depth**	3.07 ± 0.24	3.29 ± 0.32	0.013
**Lens thickness**	4.49 ± 0.26	4.52 ± 0.27	0.663
**Mean keratometry**	42.91 ± 1.62	43.06 ± 1.65	0.737
**Astigmatism**	-0.33 ± 0.17	-0.51 ± 0.27	0.014
**Pupil size**	4.06 ± 0.55	4.55 ± 0.68	0.011
**Corneal diameter**	11.64 ± 0.58	11.79 ± 0.41	0.275
**High order aberration**	0.07 ± 0.02	0.07 ± 0.02	0.918
**Spherical aberration**	0.02 ± 0.01	0.02 ± 0.01	0.334
**Subject refractive error**	-0.12 ± 0.14	-0.12 ± 0.14	0.910

UNVA = uncorrected near visual acuity. Data are mean ± standard deviation.

## Discussion

Patients undergoing Vivity implantation showed favorable uncorrected visual outcomes, especially for UDVA and UIVA. The mean UNVA was 0.22 ± 0.11 in this cohort, a value that enables patients to lead a glasses-free daily life but may not always satisfying for comfortable reading. We found that eyes with small pupils have advantages for UNVA, even with the same sSE as eyes with larger pupils, suggesting that patients with small pupils are good candidates for Vivity IOL implantation.

The variation seen in UNVA after monofocal IOL implantion has been constantly observed; it is possible that this variation is associated with the pinhole effect created by a small pupil [8] or the pseudoaccommodative effect resulting from increased corneal SA [9–12] and coma aberrations [13–16]. We found that the UNVA in emmetropic Vivity-implanted eyes is mostly affected by the pupil size. Apart from the positive effect of a small pupil on UNVA for a monofocal IOL, small pupil size has been shown to have a negative effect in certain MIOL-implanted eyes, such as a decentered MIOL [17] or a refractive MIOL [18–21]. For a diffractive MIOL, the effect of pupil size on visual function varies according to the optic design [22–25]. Although a small pupil size can have no effect, or even a negative impact, on diffractive MIOLs or refractive MIOLs, it has a positive impact on the Vivity IOL in terms of UNVA. In addition, we found no inverse relationship between pupil size and other visual functions, such as contrast sensitivity and distant or intermediate visual acuity. We speculate that a relatively small plateau—about 1 μm height at the central 2 mm of the IOL—could yield enough area for both near and distant vision. Arrigo et al., reported that eyes with larger pupil showed worse Quality of Vision score, which was related with amount of glare and halo [2]. Our data further support the potential disadvantage of Vivity IOL in eyes with larger pupil, in terms of near vision.

Unlike previous studies, we did not find a significant relation of UNVA to corneal SA [10–13] and coma [13–16]. We speculate that the pronounced negative asphericity of the Vivity IOL would offset the effect of SA in these patients, as was shown in an experiment by ReArrigo et al [2]. The eyes with an excellent uncorrected near vision, which was comparable with diffractive MIOL, had distinct features: smaller ACD and astigmatism in addition to smaller pupils.

Mini-monovision or micro-monovision has been used to compensate for the relatively small power of extended depth-of-focus IOLS. Newsom and Potvin reported favorable results of −0.75 D monovision in the nondominant eye, in terms of improved near vision, increasing the rate of glasses independence [26]. In our study, eyes with an sSE lower than -0.50 D had poorer UDVA than other eyes, although there was no significant gain in UNVA compared with eyes that had an sSE between -0.50 and 0.00 D. These data suggest that micro-monovision within -0.50 D would result in a favorable outcome for UDVA and UNVA. Further study of patient satisfaction is required to confirm this finding.

The new wavefront-shaped Vivity MIOL provides good distant and intermediate visual acuity, and it provides UNVA adequate for daily life. Patients with small pupils might benefit from the pinhole effect that can enhance the wavefront stretching effect.

## Supporting information

S1 Data (XLS)
